# COVID-19 convalescent plasma boosts early antibody titer and does not influence the adaptive immune response

**DOI:** 10.1172/jci.insight.167890

**Published:** 2023-04-24

**Authors:** John F. McDyer, Mahzad Azimpouran, Valerie L. Durkalski-Mauldin, Robert G. Clevenger, Sharon D. Yeatts, Xutao Deng, William Barsan, Robert Silbergleit, Nahed El Kassar, Iulia Popescu, Dimiter Dimitrov, Wei Li, Emily J. Lyons, Sophia C. Lieber, Mars Stone, Frederick K. Korley, Clifton W. Callaway, Larry J. Dumont, Philip J. Norris, for the SIREN-C3PO Investigators

**Affiliations:** 1 University of Pittsburgh, Pittsburgh, Pennsylvania, USA.; 2 Vitalant Research Institute, San Francisco, California, USA.; 3 Medical University of South Carolina, Charleston, South Carolina, USA.; 4 Department of Laboratory Medicine, UCSF, San Francisco, California, USA.; 5 Department of Emergency Medicine, University of Michigan Medical School, Ann Arbor, Michigan, USA.; 6 National Heart, Lung, and Blood Institute (NHLBI), NIH, Bethesda, Maryland, USA.; 7 University of Colorado School of Medicine, Aurora, Colorado, USA.

**Keywords:** COVID-19, Immunology, Adaptive immunity, Cellular immune response, Immunotherapy

## Abstract

Multiple randomized, controlled clinical trials have yielded discordant results regarding the efficacy of convalescent plasma in outpatients, with some showing an approximately 2-fold reduction in risk and others showing no effect. We quantified binding and neutralizing antibody levels in 492 of the 511 participants from the Clinical Trial of COVID-19 Convalescent Plasma in Outpatients (C3PO) of a single unit of COVID-19 convalescent plasma (CCP) versus saline infusion. In a subset of 70 participants, peripheral blood mononuclear cells were obtained to define the evolution of B and T cell responses through day 30. Binding and neutralizing antibody responses were approximately 2-fold higher 1 hour after infusion in recipients of CCP compared with saline plus multivitamin, but levels achieved by the native immune system by day 15 were almost 10-fold higher than those seen immediately after CCP administration. Infusion of CCP did not block generation of the host antibody response or skew B or T cell phenotype or maturation. Activated CD4^+^ and CD8^+^ T cells were associated with more severe disease outcome. These data show that CCP leads to a measurable boost in anti–SARS-CoV-2 antibodies but that the boost is modest and may not be sufficient to alter disease course.

## Graphical abstract

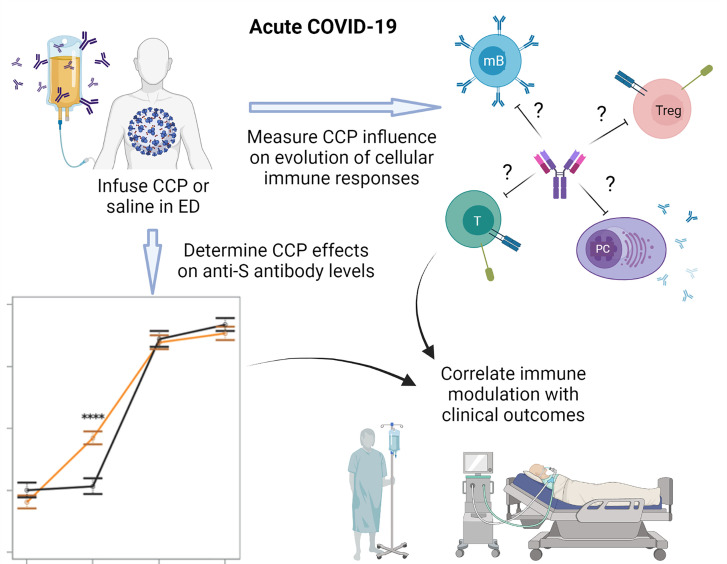

## Introduction

Antibody-based therapies have been shown to be effective treatment for COVID-19, particularly anti–SARS-CoV-2 mAb preparations, which can be given in high doses (1). The weakness of mAb therapeutics is the relatively rapid mutation rate of SARS-CoV-2 (2), with multiple mAb therapeutics now rendered ineffective against circulating strains of the virus (3, 4). In theory, use of contemporary COVID-19 convalescent plasma (CCP) or hyperimmune globulin obtained from donors who recovered from COVID-19 in the prior 6 months would provide protection from severe disease and death and would target the contemporary circulating virus. Clinical trials using CCP plasma for COVID-19 have shown variable results. Most randomized clinical trials showed no benefit of CCP in hospitalized or critically ill patients (5–9), though some trials of hospitalized patients suggested benefit (10, 11). Two randomized trials showed a benefit from early receipt of CCP in outpatients, with approximately a 50% reduction in risk of hospitalization or disease progression in trials in Argentina and the Convalescent Plasma to Limit SARS-CoV-2 Associated Complications (CSSC-004) trial in the United States (12, 13). A trial in the Netherlands was halted early after high vaccination levels in the population were achieved, but it showed a trend toward a reduction in hospitalizations after receipt of CCP with an effect size consistent with the Argentinian and CSSC-004 trials (14). In contrast, 2 randomized trials, the Clinical Trial of COVID-19 Convalescent Plasma of Outpatients (C3PO) trial in the United States and the CON-VERT trial in Spain showed no benefit of CCP in similar populations (15, 16). Preprint data from a meta-analysis suggest that, in aggregate, there is a 30% relative risk reduction for hospitalization after CCP treatment of patients with acute COVID-19 (17).

The C3PO trial enrolled participants who presented to the emergency department (ED) with acute COVID-19. The C3PO trial showed no significant benefit of CCP in preventing the primary outcome, defined as a composite of hospital admission for any reason, the seeking of emergency care or urgent care (UC), or death without hospitalization within 15 days following randomization (15). Prior studies have suggested better outcomes associated with hyperimmune i.v. immunoglobulin (IVIG) therapy in recipients who were seronegative compared with those who were seropositive prior to IVIG administration (18). The original publication of the C3PO trial results did not report baseline (BL) antibody levels prior to randomization (16). It is possible that CCP provides more benefit when administered to seronegative patients (19). In this current study of the C3PO trial, the levels of binding and neutralization antibodies were measured at 4 time points: BL (preinfusion), 1 hour after infusion, day 15, and day 30. In addition, peripheral blood mononuclear cells (PBMCs) were collected for a subset of 70 C3PO participants. Evolution of B and T cell responses was measured using flow cytometry to identify B cell maturation status, T cell activation, Treg levels, and SARS-CoV-2–specific T cell responses. We tested the hypotheses that BL seropositivity or change in antibody levels with CCP administration were associated with clinical outcome and that CCP altered the endogenous antibody response during COVID-19.

## Results

### CCP antibody levels and responses to CCP administration.

**Figure 1:**
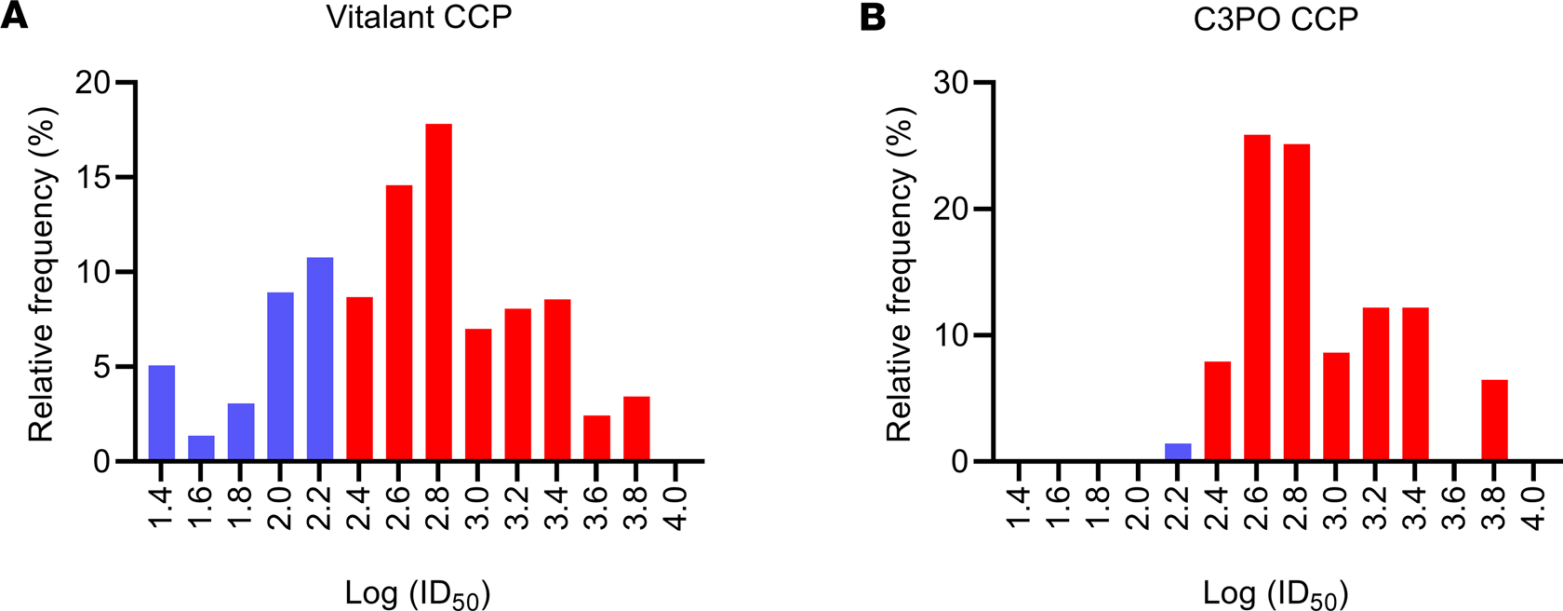
Convalescent plasma neutralization titer distribution. (**A**) Screening of ID_50_ neutralization titers was performed at the Broad Institute for 1,598 donors who met clinical criteria for convalescent plasma donation. (**B**) The distribution of neutralization titers is shown for the 139 convalescent plasma units used to make doses that were transfused in the C3PO trial. Red bars on each graph denote units that met the FDA guideline threshold of ID_50_ ≥ 1:250 for designation of high-titer convalescent plasma, and blue bars denote units that fell below the threshold.

As previously reported, CCP was collected per FDA guidelines and was tested using a live-virus plaque reduction neutralization test (PRNT) at the Broad Institute (15). A 50% inhibitory dilution (ID_50_) of 1:250 was considered high titer by the FDA and eligible for transfusion as CCP. Screening of 1,598 donors eligible to donate CCP revealed that 66% met the threshold of an ID_50_ ≥ 1:250 using the Broad Institute assay (Figure 1A). In total, 139 collections from 128 unique donors were used to create doses of CCP infused to recipients in the C3PO trial, and the median titer of infused CCP units was 1:578 (IQR, 1:445–1:1,692; Figure 1B).

**Figure 2:**
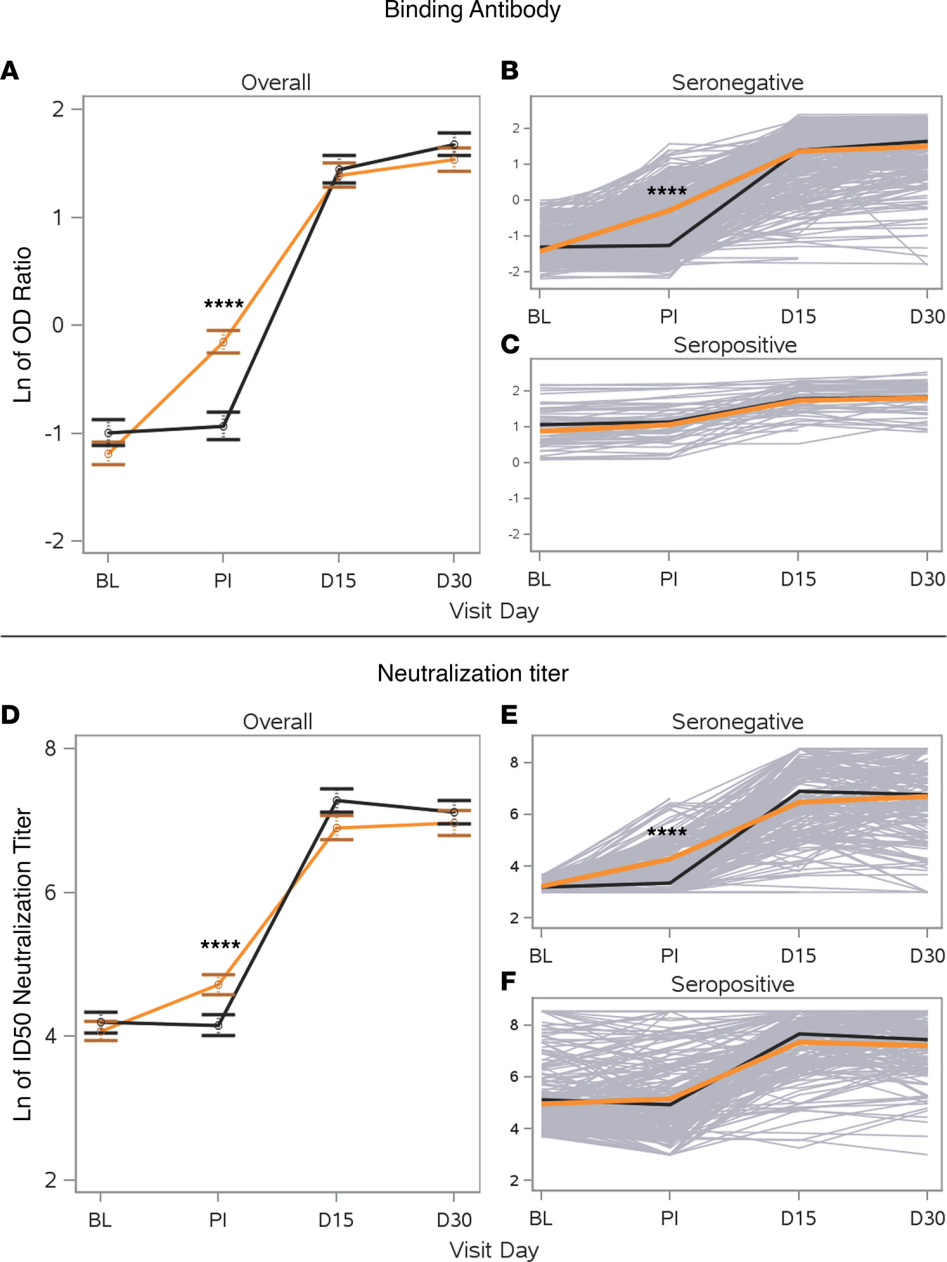
Evolution of antibody responses in study participants. (**A**) Binding antibody responses were measured using an ELISA from EUROIMMUN, and the natural log of the ratio of the sample OD to a calibrator OD is shown for the saline (black line) versus CCP (orange line) recipients across the 4 sample time points. (**B** and **C**) Individual participants’ data are shown in gray lines, with the average for the saline (black) and CCP (orange) recipients plotted for subjects who were seronegative for binding antibodies at BL (**B**) or seropositive for binding antibodies at BL (**C**). (**D**) Neutralizing antibody activity was measured using the Broad Institute assay and reported as the 50% inhibitory dose (ID_50_). (**E** and **F**) Results separated by participants who were seronegative (**E**) or seropositive (**F**) at BL for neutralizing antibody activity (titer threshold of 1:40). Error bars represent 95% CI limits for the mean. ****P < 0.0001 for saline versus CCP groups at each time point. *P* values are based on a 2-tailed *t* test comparing the differences within each specific visit day, adjusted for multiple comparisons.

**Figure 3:**
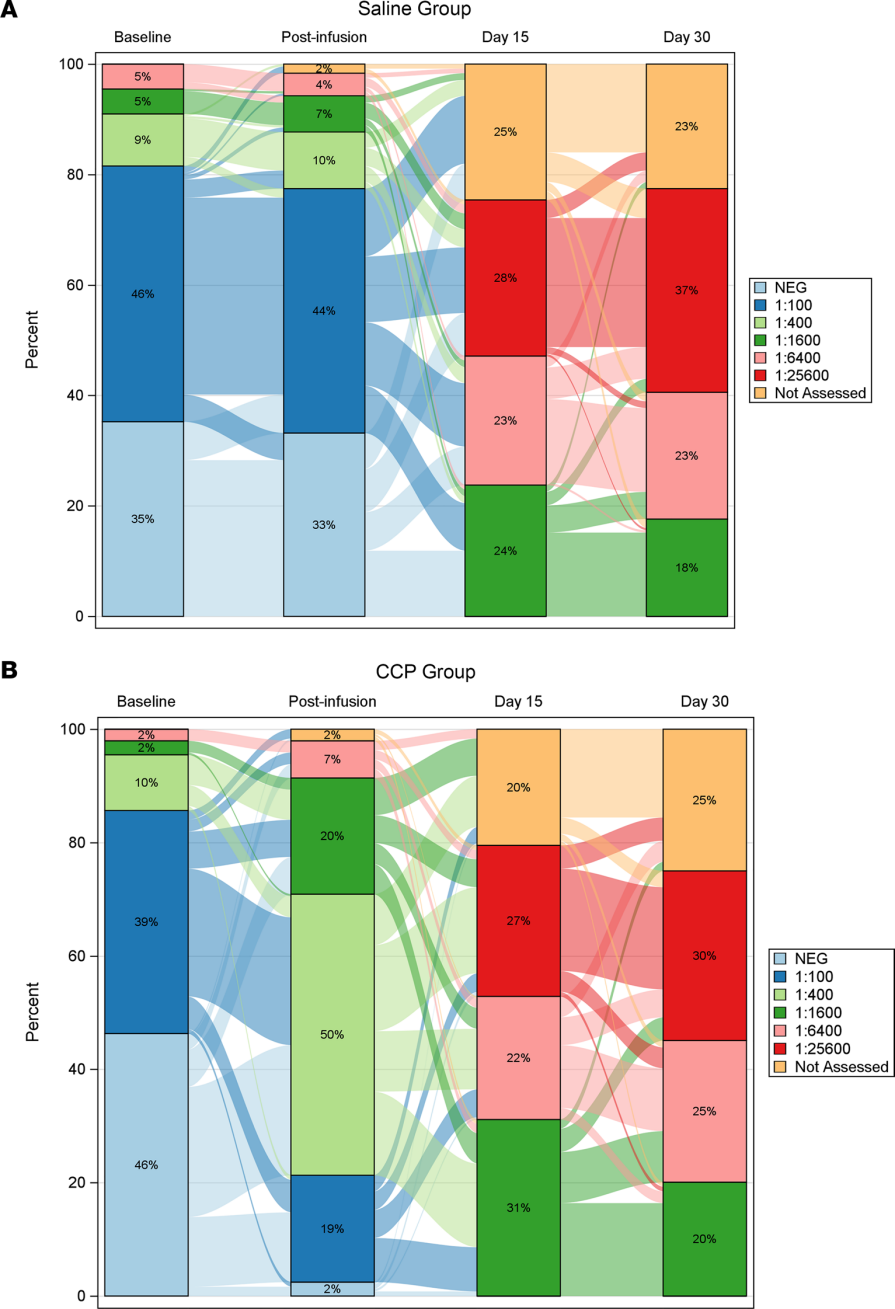
Evolution of RBD binding antibody titer in recipients of saline versus CCP. RBD binding antibody titers were calculated using endpoint dilutions, with positive values defined as titer ≥ 1:400. (**A** and **B**) Sankey diagrams illustrate the proportion of subjects with a given antibody titer at each time point, and their flow to the next titer is indicated by shaded lines between the bars representing each time point for saline and CCP recipients.

Binding and neutralizing antibody levels were measured at BL (preinfusion), after infusion (PI; 1 hour), day 15, and day 30. CCP recipients had almost a 3-fold increase in SARS-CoV-2 spike binding antibody levels from BL to PI, and the PI levels were significantly higher in the CCP compared to saline with multivitamin arm, hereafter referred to as “saline” (Figure 2A). This difference was significant in participants who were seronegative at BL (Figure 2B) but not in those who were seropositive at BL (Figure 2C). There was considerable dilution of the CCP product after infusion, which can be seen by comparison of the titer in CCP with the paired titer PI in the recipients (Supplemental Figure 1; supplemental material available online with this article; https://doi.org/10.1172/jci.insight.167890DS1), particularly for those who were seronegative at BL (Supplemental Figure 1A). By day 15, spike-specific antibody levels were significantly higher compared with BL/PI levels in both the CCP-treated group and saline group, with the increase in levels much larger than those seen 1 hour after infusion of CCP. Increased spike-specific antibody levels were similar at day 15 versus day 30 in both groups, consistent with a peak in the endogenous spike antibody response. Despite only a modest correlation between spike binding antibody levels and neutralization titer (Spearman’s ρ = 0.35; Supplemental Figure 2), similar results were obtained when the neutralizing antibody titer was measured longitudinally in recipients. CCP recipients showed a 1.9-fold increase in ID_50_ titer from BL to PI, and the level PI was significantly higher than in the saline arm (Figure 2D). As with binding antibodies, the increase in neutralization titer from BL to day 15 was much greater than that induced by infusion of CCP. A significant difference in neutralizing antibody titer between the saline and CCP groups at the PI time point was again seen in the participants who were seronegative but not in those who were seropositive at BL (Figure 2, E and F). Geometric mean titers are shown in Supplemental Table 2. The proportion of participants PI who had a titer ≤ 1:100 on an in-house receptor-binding domain (RBD) binding antibody ELISA was 77% in saline recipients (Figure 3A) versus 21% in CCP recipients (Figure 3B). Together, these data show that CCP infusion had a significant impact on spike-specific antibody and neutralizing antibody activity, but they show that this increase was modest compared with levels generated by the host immune system by day 15 and did not significantly affect binding or neutralizing antibody responses in participants who were seropositive prior to CCP infusion.

### Effect of BL serostatus on disease outcome.

The C3PO trial showed no significant benefit of CCP in preventing the primary outcome, disease progression defined as seeking ED or UC, hospitalization, or death within 15 days (15). Prior studies have suggested better outcomes associated with hyperimmune IVIG therapy in recipients who were seronegative compared with those who were seropositive prior to IVIG administration (18). We examined whether BL serostatus was associated with modification of risk of disease progression in recipients of CCP. Using the RBD binding antibody assay, 41% of trial participants were seronegative upon presentation. Similarly, 49% of participants showed a neutralization titer < 1:40 at BL. The unadjusted risk difference of disease progression for each assay had wide confidence intervals and did not differ depending on the participant’s BL serostatus (Table 1). These results show that, within the C3PO trial population, BL serostatus did not influence the treatment effect of CCP.

### Cellular immunology study population.

Seventy participants in the C3PO study were enrolled in a substudy to measure the evolution of B and T cell responses and to determine if they were influenced by receipt of CCP. Enrollment was performed at the 6 medical centers with the highest projected enrollment and ability to ship whole blood samples overnight to San Francisco, California, USA, after collection. Participants included 38 males and 32 females, with a mean age of 52 (range, 20–78). Among these individuals, 35 received CCP and 35 received normal saline plus multivitamins. Fourteen individuals were hospitalized, including 1 who was mechanically ventilated and required additional organ support. Details on the demographic and clinical characteristics of all participants are shown in Table 2.

### Evolution of B and T cell responses.

**Figure 4:**
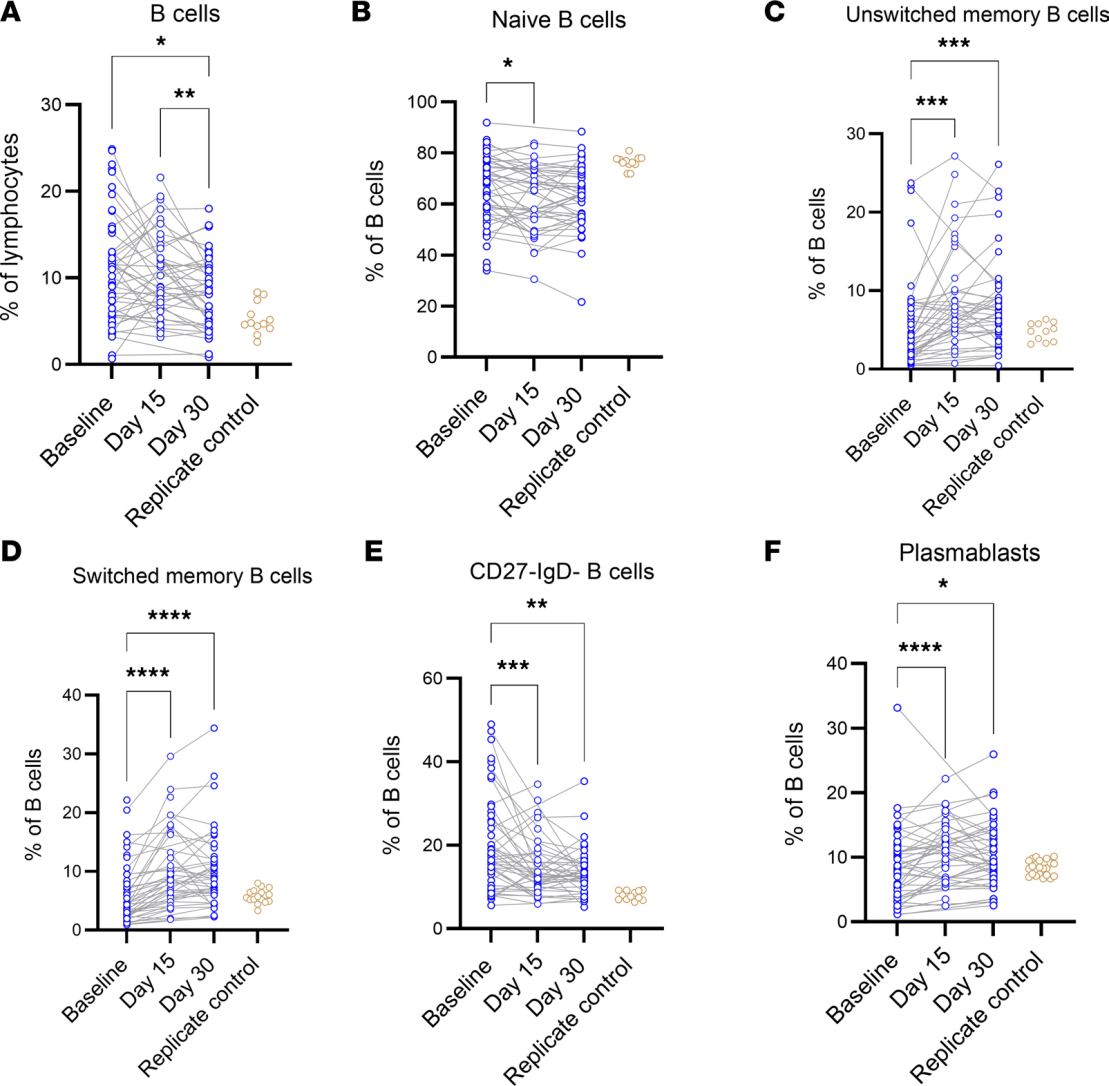
Evolution of B cell responses. (**A**–**F**) CCP and saline populations were combined to measure B cell phenotypes across 3 time points in all subjects with PBMC samples: BL, day 15, and day 30 for B cells, plasmablasts, switched memory B cells, naive B cells, CD27^–^IgD^–^ B cells, and unswitched memory B cells. The replicate control group on each graph represents a replicate aliquot of a single healthy control run with each batch on the flow cytometer. Time points were compared with a mixed-effects model with a Šidák’s multiple comparison test. *P < 0.05, **P < 0.01, ***P < 0.001, ****P < 0.0001.

We first looked at the evolution of B cell populations at BL, day 15, and day 30). B cells were gated as CD19^+^, with CD3, CD14, and CD56 used as dump parameters to eliminate T cells, monocytes, and NK cells, respectively. Plasmablasts were gated as CD38^+^ and CD27^+^ B cells (20). CD38^–/int^ cells were stained for IgD and CD27 expression. IgD^–^ and CD27^+^ cells were defined as switched memory B cells, and IgD^+^CD27^+^ was defined as unswitched memory B cells. IgD^+^CD27^–^ cells represented naive B cells (Supplemental Figure 3). Plasmablasts and unswitched memory B cell populations showed an increase by day 15 compared with BL (*P* = 0.043 and 0.034, respectively). Switched memory B cells showed an increase at days 15 and 30 compared with BL (*P* = 0.040 and 0.010, respectively) (Figure 4). These results show that CCP does not impair the maturation of B cells after SARS-CoV-2 infection. In addition, the plasmablast response is not blunted in CCP compared with saline recipients.

**Figure 5:**
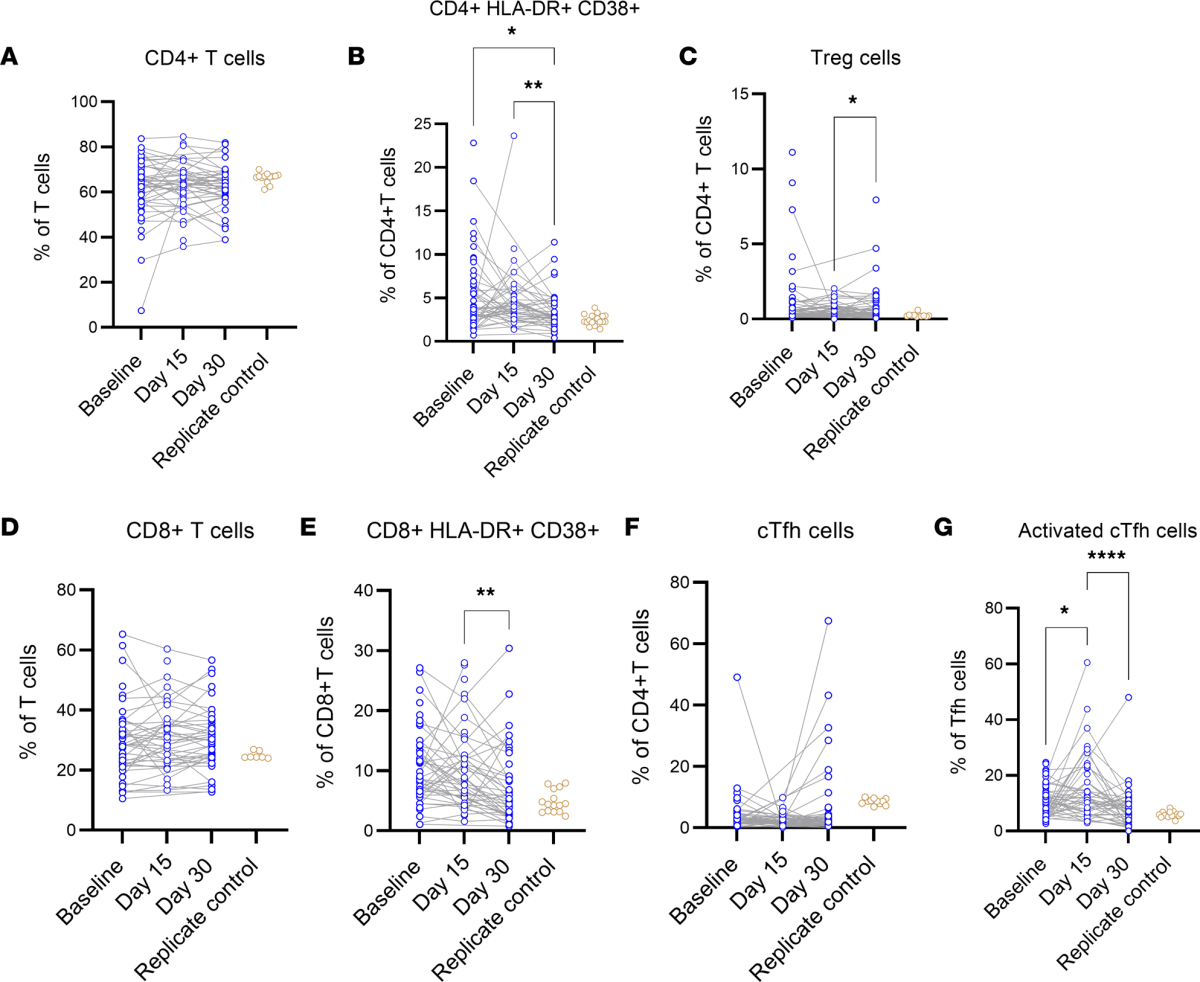
T cell phenotype evolution. (**A**–**G**) CCP and saline populations were combined to measure T cell phenotypes across 3 time points in all subjects with PBMC samples: BL, day 15, and day 30 for CD4^+^ T cells, CD4^+^HLA-DR^+^CD38^+^ T cells, Tregs, CD8^+^ T cells, CD8^+^HLA-DR^+^CD38^+^ T cells, cTfh cells, and activated cTfh cells. The replicate control group on each graph represents a replicate aliquot of a single healthy control run with each batch on the flow cytometer. Time points were compared with a mixed-effects model with a Šidák’s multiple comparison test. *P < 0.05, **P < 0.01, ****P < 0.0001.

**Figure 6:**
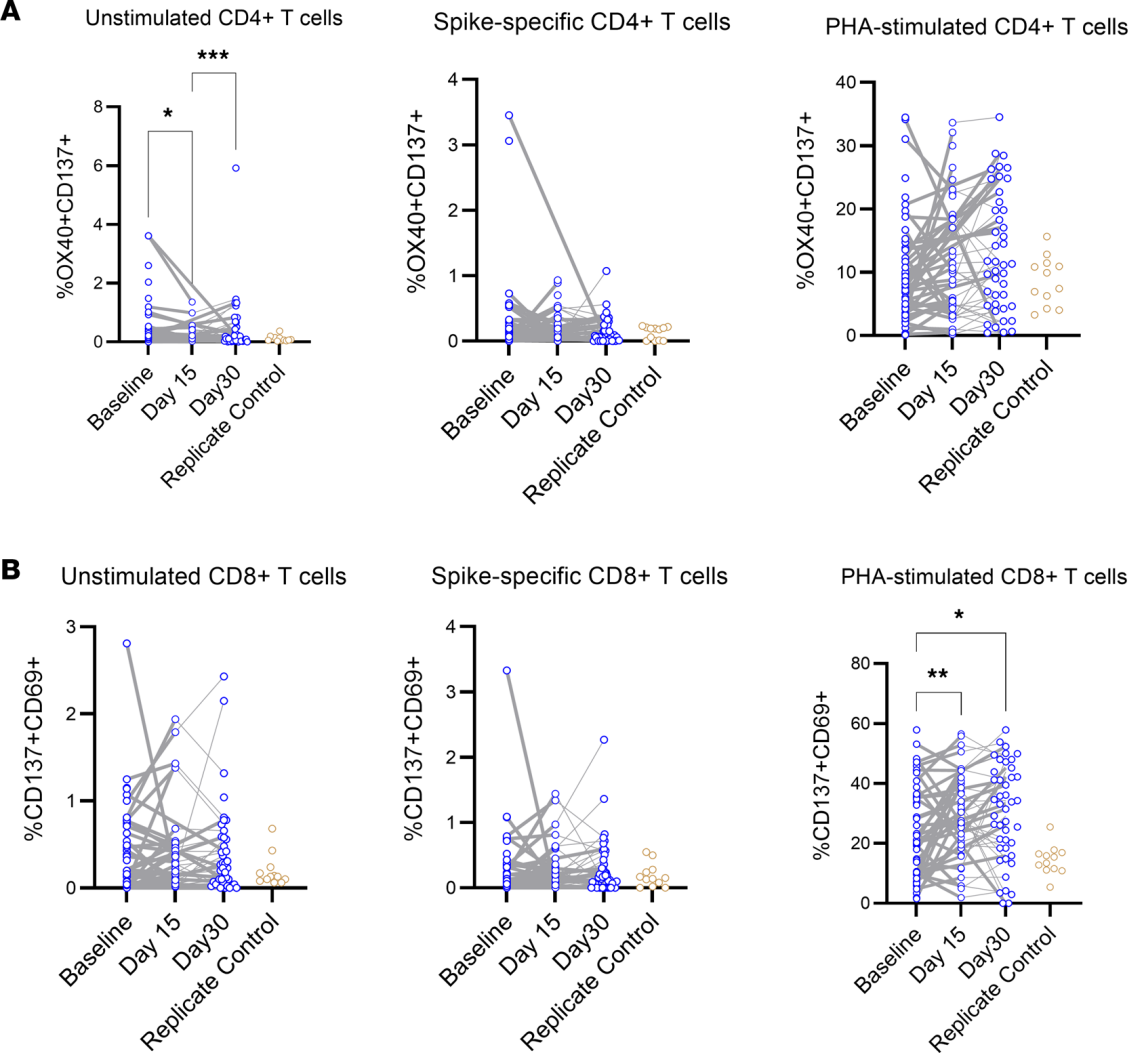
Frequency of SARS-CoV-2–specific T cells. (**A** and **B**) CCP and saline populations were combined to measure T cell phenotypes across 3 time points in all subjects with PBMC samples: BL, day 15, and day 30 for CD4^+^ (**A**) and CD8^+^ (**B**) T cells. Spike-specific T cells were defined as the percentage of positive cells in the peptide-stimulated minus unstimulated populations. Time points were compared with a mixed-effects model with a Šidák’s multiple comparison test. *P < 0.05, **P < 0.01, ***P < 0.001.

CD4^+^ Tregs were defined by Foxp3 versus CD25 expression. CD4^+^CXCR5^+^ circulating T follicular helper (cTfh) cells were plotted as ICOS^+^ versus CD38^+^ to gate activated cTfh2. Activated CD4^+^ and CD8^+^ T cells were identified by CD137 versus OX40 and CD69 expression, respectively (Supplemental Figure 4) (21). CD4^+^HLA-DR^+^CD38^+^ T cells were lower on day 30 than at baseline or day 15, and activated cTfh showed a peak at day 15 (Figure 5). Antigen-specific T cells were identified by coexpression of CD137 and OX40 for CD4^+^ T cells and coexpression of CD137 and CD69 for CD8^+^ T cells after stimulation with a SARS-CoV-2 spike megapool of peptides (22) or phytohemagglutinin (PHA) as a positive control (Supplemental Figure 5). There was no change in the level of spike-specific T cells detected over 30 days for CD4^+^ or CD8^+^ T cells (Figure 6, A and B). CD8^+^ T cells showed an increase in cells responding to PHA at days 15 and 30 compared with BL (Figure 6B).

### Correlation of cellular immune responses with receipt of CCP and with disease outcome.

**Figure 7:**
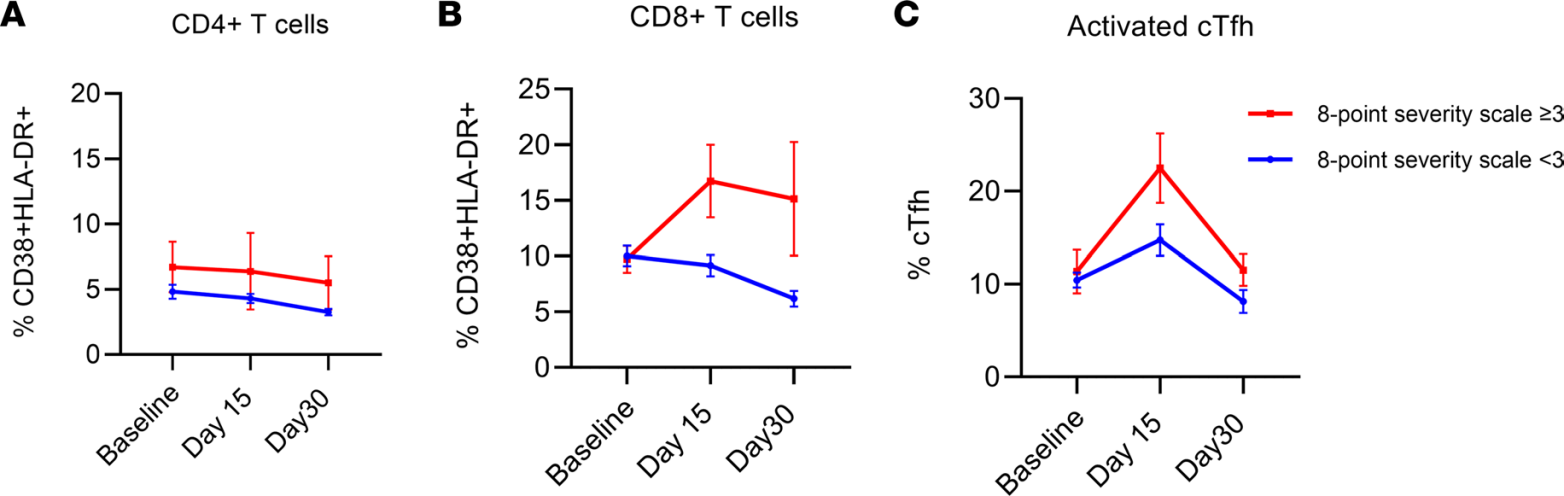
Correlation between disease severity and activated T cell populations. All 22 cellular immune phenotypes were analyzed for association with disease outcome, with the 8-point severity scale categorized as < 3 or ≥ 3. Subjects who reported ≥ 3 symptoms on an 8-point severity scale are represented in red, and those with < 3 symptoms in blue. (**A**–**C**) Populations that showed increased levels in patients with score ≥3 included CD4^+^CD38^+^HLA-DR^+^ T cells (**A**), CD8^+^CD38^+^HLA-DR^+^ T cells (**B**), and activated cTfh cells (**C**), which were CD3^+^CD4^+^CD45RA^–^CXCR5^+^CD38^+^ICOS^+^ cells. Comparisons were made using a linear mixed-effect model to determine significant associations. Data are shown as mean ± SEM.

We next looked at differences in immune profiles between CCP and saline recipients. There was no significant association between any of the cellular phenotypes measured and receipt of CCP (Supplemental Figure 6). We also looked at cellular phenotypes based on the primary outcome (death, hospitalization, or ED/UC visit within 15 days after randomization), disease progression on the COVID-19 outpatient ordinal scale within 15 days (symptom worsening), and 8-point illness severity scale ≥ 3. The 8-point scale was adapted from a February 2020 WHO COVID-19 Ordinal Scale for Clinical Improvement, where a score of 1 is asymptomatic, ≥ 3 is hospitalized, and 8 is death (23). There was a significant correlation between worst COVID-19 outcome on the 8-point scale and activated cTfh cells (*P* = 0.005) and between worst COVID-19 outcome on the 8-point scale and both CD38 and HLA-DR upregulation on CD4^+^ T cells (*P* = 0.008) and CD8^+^ T cells (*P* = 0.002) (Figure 7). These results demonstrate that CCP does not decrease the proinflammatory environment of acute COVID-19 but that increased T cell activation is associated with more severe symptoms in an outpatient population.

### Antibody levels in C3PO trial recipients were not correlated with B or T cell phenotypes.

We explored the correlation between antibody levels and cellular immune parameters using Spearman’s correlation. We first examined whether antibody levels correlated with cellular immune responses at each time point (BL, day 15, and day 30) as well as whether cellular responses correlated with the change in antibody response from BL to day 15 or day 30. None of the cellular phenotypes showed a significant correlation with concurrent antibody levels or change in antibody levels.

## Discussion

We found that infusion of 1 unit of high-titer CCP significantly increased anti–SARS-CoV-2 RBD-specific IgG, anti–SARS-CoV-2 spike IgG, and neutralizing antibody levels in recipients. Despite these increases in antibody levels, we did not observe a significant difference in hospitalization within 28 days, the primary outcome of the C3PO trial. Moreover, levels achieved after infusion of CCP were significantly lower compared with endogenously generated antibody levels at days 15 or 30, suggesting that despite high-titer CCP, the impact on circulating antibody levels was modest. Our findings indicate that CCP administration did not negatively impact the host antibody response to SARS-CoV-2, as the day 15 and 30 levels did not differ between the CCP and saline arms. Additionally, detailed examination of cellular immune responses showed that B and T cell maturation were not affected by CCP administration. Finally, we found that CD4^+^ and CD8^+^ T cell activation was associated with more severe disease outcome.

We observed that those with lower anti-spike antibody titers at BL had the most significant boost in RBD-specific antibody levels; thus, those with higher titer BL levels of RBD-specific antibody appear to have derived the least immune impact from CCP administration. Nonetheless, when analysis was limited to individuals who were seronegative at baseline, CCP receipt was not associated with significant clinical benefit. Thus, the CCP-induced increase in antibodies was insufficient to alter the clinical course of COVID-19, including in seronegative patients. Retrospective studies suggest that CCP improves survival in hematological malignancies (24, 25), and a randomized, controlled trial found improved survival in cancer patients who received CCP (26).

It has been shown that anti–SARS-CoV-2 mAb therapy is an effective therapy in preventing severe COVID-19 or death, at least in virus variants that have not yet escaped the antibody sequence. The level of anti–SARS-CoV-2 antibody delivered with mAb therapy is higher than that contained in even high-titer CCP. While it is difficult to compare antibody values across assays, the FDA lists high-titer CCP as ≥ 1,280 AU/mL for the Abbott ARCHITECT assay and > 55 RU/mL for the EUROIMMUN assay used in our study. Anti–S IgG levels within 48 hours after infusion of 1,200 mg REGN-COV mAb cocktail ranged from > 80,000 to 332,000 arbitrary units (AU)/mL using the Abbott ARCHITECT assay, which are orders of magnitude higher than the FDA cutoff for high-titer CCP (27). The median level observed after CCP administration using the EUROIMMUN assay was 0.78 RU/mL, almost 2 orders of magnitude below the FDA threshold for high-titer CCP. Consistent with mAbs delivering a much higher dose of antibody, SARS-CoV-2–specific antibody levels declined 60% by day 12 after mAb infusion (27), while they increased substantially by day 15 in our study of CCP recipients.

Four recent randomized, controlled trials of CCP in outpatients were evenly split between those showing efficacy (12, 13) and those showing no effect (15, 16), with a fifth showing potential efficacy that did not reach statistical significance (14). There were some differences in trial design, such as the positive CSSC-004 and Dutch trials using pre–COVID-19 plasma rather than normal saline as a placebo (13, 14), which could favor CCP if the plasma itself were associated with adverse outcomes, or C3PO enrolling subjects presenting to the ED (15), which may bias toward participants too far advanced in their disease course to benefit from CCP. However, the Argentinian CCP trial used normal saline as the placebo and still showed the same positive effect size as the CSSC-004 trial (12), implying that using plasma in the control arm was not responsible for the positive effect seen in that trial. Conversely, the CON-VERT study in Spain was performed using community participants rather than those presenting to the ED and showed no effect of CCP (16), implying that recruiting subjects in the ED alone does not entirely explain why the C3PO trial did not show a treatment effect for CCP. The titer of CCP administered across these 4 trials was marginally lower in the positive efficacy trials compared with the negative trials, though the same neutralization assay was not used to test samples from the different trials (17); this is evidence that the dose of antibody was not the sole determinate of outcome in the CCP trials. Recent data suggest that N-specific Fc-mediated antibody maybe an important effector pathway for CCP (28), and in mouse models, CCP with low neutralization activity can still provide some protection from SARS-CoV-2 through Fc-mediated functions such as antibody dependent cellular cytotoxicity (29).

Our data show that activated CD4^+^ and CD8^+^ T cells were associated with more severe disease outcome on an 8-point scale but that receipt of CCP did not affect B or T cell population phenotypes. We hypothesized that receipt of CCP might have blunted the host anti-S antibody response, in part due to historical information such as the use of anti-D antibody to block Rh immunization (30). Two studies of mAb therapy suggest modest suppression of host anti-spike antibody responses, either through monitoring of antibodies targeting regions of spike that are not targeted by the mAb (31) or via measuring anti–spike IgM in patients versus controls treated with mAb therapy (32). In contrast, a study of CCP showed no difference in anti-spike antibody levels within days after therapy between those who did or did not receive CCP (14), consistent with our findings. It is tempting to speculate that the dose of anti-spike antibody administered in CCP is not sufficient to blunt host B cell responses.

While the current study draws on the strength of examining samples from a controlled, randomized trial, there are several limitations. Study of early evolution of antibody and cellular immune responses inside the 2-week window were not possible, given the timing of sample collection. Binding and neutralizing capability of antibody responses were quantified, but other antibody-driven functions such as antibody-dependent cellular cytotoxicity were not studied. Examination of cellular immune responses was performed by flow cytometry, and differences in minor populations of immune cells or other functional responses could exist that would be detected by in-depth examination with techniques such as single-cell RNA-Seq.

In summary, we found that infusion of CCP raised anti–SARS-CoV-2 antibody levels modestly immediately after infusion, but to a much lower level than that achieved by the host immune response at day 15. Receipt of CCP did not blunt the native immune response at the level of antibody titer or T or B cell maturation state. Participants who had lower BL levels of anti–SARS-CoV-2 antibodies experienced a greater increase in antibody titer after CCP infusion, but the clinical outcome was not improved in this subset of participants compared with the overall trial population. When we compared the antibody levels achieved in C3PO to similar randomized trials of CCP in outpatients, we could not identify a difference that would account for discrepant trial results. One possible interpretation of the multiple clinical trials would be that the overall dose of anti–SARS-CoV-2 antibody contained in a unit of CCP is low compared with that achieved using mAb preparations or generated by the immune system after natural infection. It is possible that higher titer CCP my produce more consistent positive results than currently defined high-titer CCP. It has been reported that plasma collected from CCP donors who had received SARS-CoV-2 vaccination possess higher neutralizing antibody activity than those who remained unvaccinated (33), and this will allow exploration of next-generation CCP for eligible patients.

## Methods

### Study population.

The C3PO trial was a phase III, multicenter, randomized, single-blind, placebo-controlled trial that enrolled ED patients presenting with mild COVID-19. Participants received either 1 unit of high-titer CCP or 250 mL of saline with multivitamins. Eligible participants were ≥ 50 years old or had 1 or more risk factors for disease progression, presented to the ED ≤ 7 days after symptom onset, and they were deemed by the clinical team stable for outpatient management without supplemental oxygen. Exclusion criteria included being ≤ 18 years old, being prisoners or wards of the state, having an inability to complete follow-up assessments, having a history of adverse reactions from transfusion, or having an inability to receive 250 mL of fluid. Patients who had received blood products within the past 120 days or another investigational treatment for COVID-19, including anti–SARS-CoV-2 mAbs or vaccination, were excluded. Enrollment ran from August 2020 through February 2021 at 48 EDs in 21 states. Participants with a BL and ≥ 1 post-BL antibody assay result were included in the current analysis.

### CCP.

CCP was collected from donors according to the FDA guidance for donor eligibility. CCP units were qualified PRNT performed by the Broad Institute. Units with an ID_50_ ≥ 1:250 were eligible to be transfused.

### Sample collection and processing.

Blood samples were obtained from consenting participants prior to study drug infusion (BL), 1 hour PI, and on days 15 and 30 following randomization. Blood samples were processed into serum and plasma, aliquoted, and stored locally at –70°C. Study samples were shipped to a central biorepository at the University of Pittsburgh and were then sent to the analytic laboratories.

### Cellular immunology substudy participants and sample collection.

A subset of 70 C3PO participants presenting to 6 high-enrolling centers in the C3PO study (Baystate Health, Cooper, Maine Medical, Michigan Medicine, Spectrum Health, and UCLA) was included for study of cellular immune responses. Samples were collected before transfusion and at days 15 and 30. Blood was collected in two 10 mL EDTA tubes and shipped overnight at ambient temperature to Vitalant Research Institute (San Francisco, California, USA). Ficoll-Paque (Lymphoprep, Stemcell Technologies) was used for the isolation of PBMCs. PBMCs were cryopreserved in FBS (heat-inactivated, Hyclone Laboratories) with 10% dimethyl sulfoxide (DMSO, MilliporeSigma) and stored in liquid nitrogen in 5 × 10^6^ cell aliquots.

### Binding antibody detection.

Anti–SARS-CoV-2 RBD antibodies were detected using an ELISA customized at the University of Pittsburgh. Ninety-six–well plates were coated with 50 μL of recombinant RBD (20 μg/mL) overnight at 4°C and were then blocked with 100 μL of skim milk for 1 hour at 4°C before being washed using 150 μL of PBS. Plasma samples were serially diluted (1:100, 1:400, 1:1,600, etc.) in skim milk, and 50 μL was added to the plate and incubated for 1 hour at 37°C. Plates were washed, and 50 μL of goat anti–human IgG HR–labeled secondary antibody (Southern Biotech, 2040-05; diluted to 1:3,000 with skim milk) was added and incubated for 1 hour at 25°C. Plates were washed, and 50 μL of TMB substrate was added and incubated for 7 minutes before adding 50 µL of stop solution (0.16M H_2_SO_4_). Plates were read at 450 nm to obtain optical density (OD) values. An OD value threshold of 0.3 was set for positivity, and this was twice the level seen in blank wells.

Total antibodies against SARS-CoV-2 S1 were measured using a commercially available ELISA (EUROIMMUN) according to the manufacturer’s directions. Assay results were reported as a ratio of OD of the clinical sample to OD of a calibrator. An OD ratio < 0.8 was considered negative, ≤ 0.8 to < 1.1 was considered borderline, and ≥ 1.1 was considered positive.

### Neutralizing antibody detection.

Neutralizing antibody levels were measured by the Broad Institute using a live-virus SARS-CoV-2 PRNT as previously described (34). Neutralizing antibody serum samples were tested at a 1:40 dilution and then serially diluted 4-fold up to 4 times before being mixed with live SARS-CoV-2 (D614G) for 1 hour. The mixture was added to Vero E6-TMPRSS2 cells for 48 hours, and then, infected cells were detected with anti–SARS-CoV-2 nucleoprotein mouse primary antibody (Sino Biological) and a secondary Alexa Fluor 488–conjugated goat anti-mouse antibody (Jackson ImmunoResearch).

### Flow cytometry analysis.

Cells were thawed in 15 mL prewarmed RPMI (Thermo Fisher Scientific); then, 1 × 10^6^ cells were resuspended in 96-well V-bottom polystyrene plates, stained with Zombie Aqua/Nir (BioLegend), and washed prior to surface staining with a surface antibody cocktail for 30 minutes at 4°C in the dark. Following surface staining, cells were washed twice with PBS plus 2% FBS. For intracellular staining, cells were resuspended with 100 μL BD Cytofix/Cytoperm for 15 minutes at 4°C in the dark and were then washed twice with BD Perm/Wash buffer and resuspended in 50 μL of BD Perm/Wash buffer containing Foxp3-PE. They were then incubated at 4°C for 30 minutes in the dark. Cells were washed twice with 250 μL BD Perm/Wash buffer and resuspended in PBS prior to flow cytometric analysis. A list of antibodies for these panels can be found in Supplemental Table 1. Samples were run on a Cytek Aurora 5-Laser cytometer. Samples were tested blinded in batches of 8–16, with 250,000 cells acquired per sample. A replicate PBMC sample from the same healthy donor was included in each batch. Data analysis was performed with SpectroFlo software.

### Measuring SARS-CoV-2–specific T cells.

Peptides spanning SARS-CoV-2 spike glycoprotein, 13 or 17 mers overlapping by 10 amino acids (*n* = 181), were obtained from BEI Resources (peptide array NR-52402), reconstituted with 25 μL DMSO, pooled, and relyophilized (35). The lyophilized megapool was resuspended in 500 μL DMSO and 1.5 mL H_2_O. Cells were incubated with of SARS-CoV-2 peptide pool (1 μg/mL) or PHA-L (1.25 mg/mL, Invitrogen) for 24 hours at 37°C, 5% CO_2_. The next day, cells were stained with Zombie Aqua (BioLegend) and for surface markers.

### Statistics.

Antibody data were analyzed using SAS V9.4 or higher (SAS Institute). Assay results were positively skewed, so they were transformed to a natural log scale for analyses. The effect of treatment on antibody levels over time was evaluated using a generalized linear model adjusting for multiple comparisons. The MIXED procedure in the SAS system was used to fit the model and included an interaction term for treatment group and visit day. Unadjusted risk differences with 95% CI are reported for the assessment of CCP’s effect on disease progression within serostatus subgroups for both the binding and neutralizing antibody assays.

Cellular immune data were analyzed using GraphPad Prism v.9.1.2. A mixed-effect analysis was performed with a Šidák’s multiple-comparison test to compare across time points, and *P* < 0.05 were considered statistically significant. We used a linear mixed-effect model to evaluate the association between outcome and cellular immune phenotypes (R LME4 package). Specifically, we used correlated random intercept and slope to generate the model, where subjects are random-effect variables, visit and outcome are fixed-effect variables, and B cell and T cell phenotype are response variables. Samples with cell viability < 90% were excluded from the analysis.

### Study approval.

The Food and Drug Administration (FDA) approved an Investigational New Drug application for the trial. A central IRB (Advarra) reviewed and approved the trial protocol for all participating sites. An independent medical safety monitor reviewed and adjudicated all serious adverse events, and the NHLBI appointed the independent data and safety monitoring board. All participants were enrolled under written informed consent.

## Author contributions

JFM, NEK, FKK, CWC, LJD, and PJN conceived of the study. WB, RS, and LJD contributed clinical samples. MA, MS, IP, DD, WL, EJL, and SCL performed experiments. VLDM, RGC, SDY, and XD performed data analysis. PJN, VLD, and JFM wrote the manuscript.

## Supplementary Material

Supplemental data

## Figures and Tables

**Table 1:**
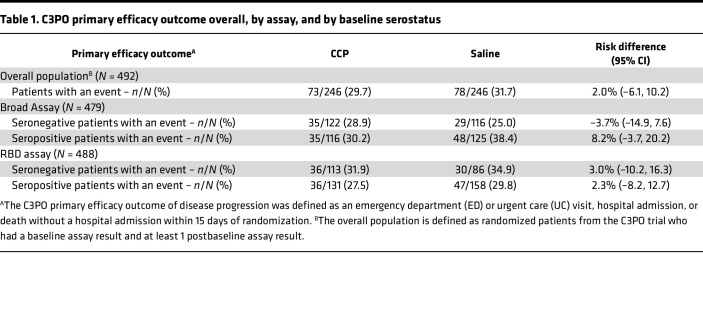
C3PO primary efficacy outcome overall, by assay, and by baseline serostatus

**Table 2:**
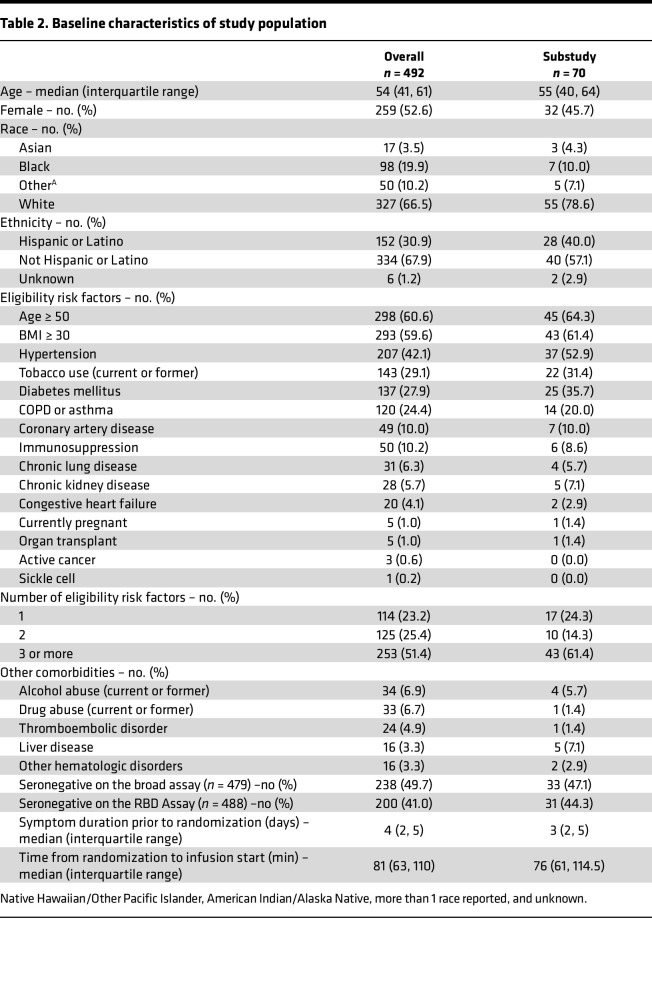
Baseline characteristics of study population

## Version 1 03/02/2023

In-Press Preview

## Version 2 04/24/2023

Electronic publication

## Version 3 05/11/2023

SIREN-C3PO Investigators was added to the author list
