# Supplementary material for: COVID-19 convalescent plasma boosts early antibody titer and does not influence the adaptive immune response
Source: JCI Insight. 2023 Apr 24;8(8):e167890. doi: 10.1172/jci.insight.167890 (PMC10174456; doi:10.1172/jci.insight.167890)
Supplement: Supplemental data [file jciinsight-8-167890-s227.pdf]

(A)

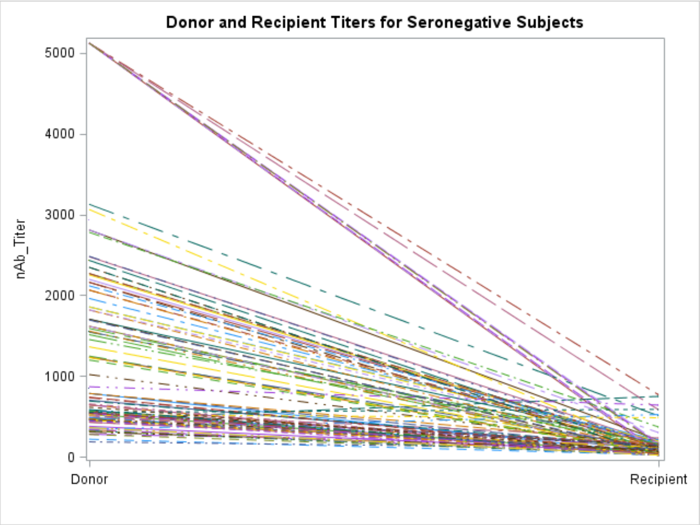

(B)

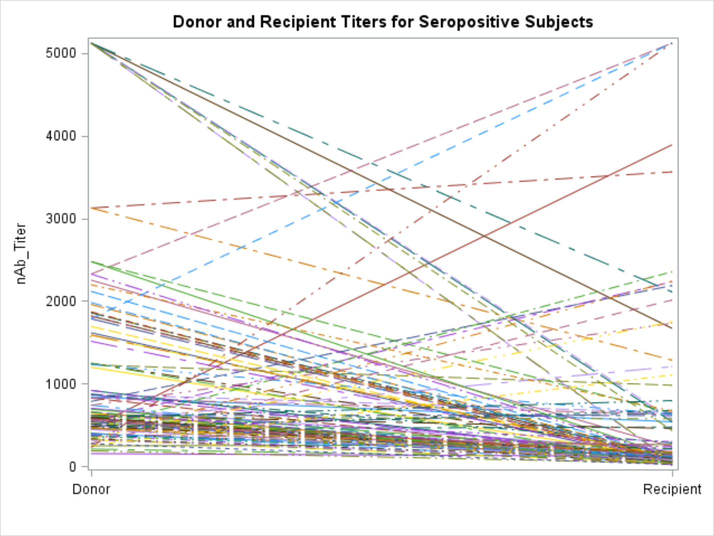

Figure S1. Comparison of neutralizing titers in CCP units to recipients after CCP administration.
Neutralization titers are shown for each CCP donor unit and the paired recipient for (A) recipients who were seronegative at baseline and (B) seropositive at baseline.

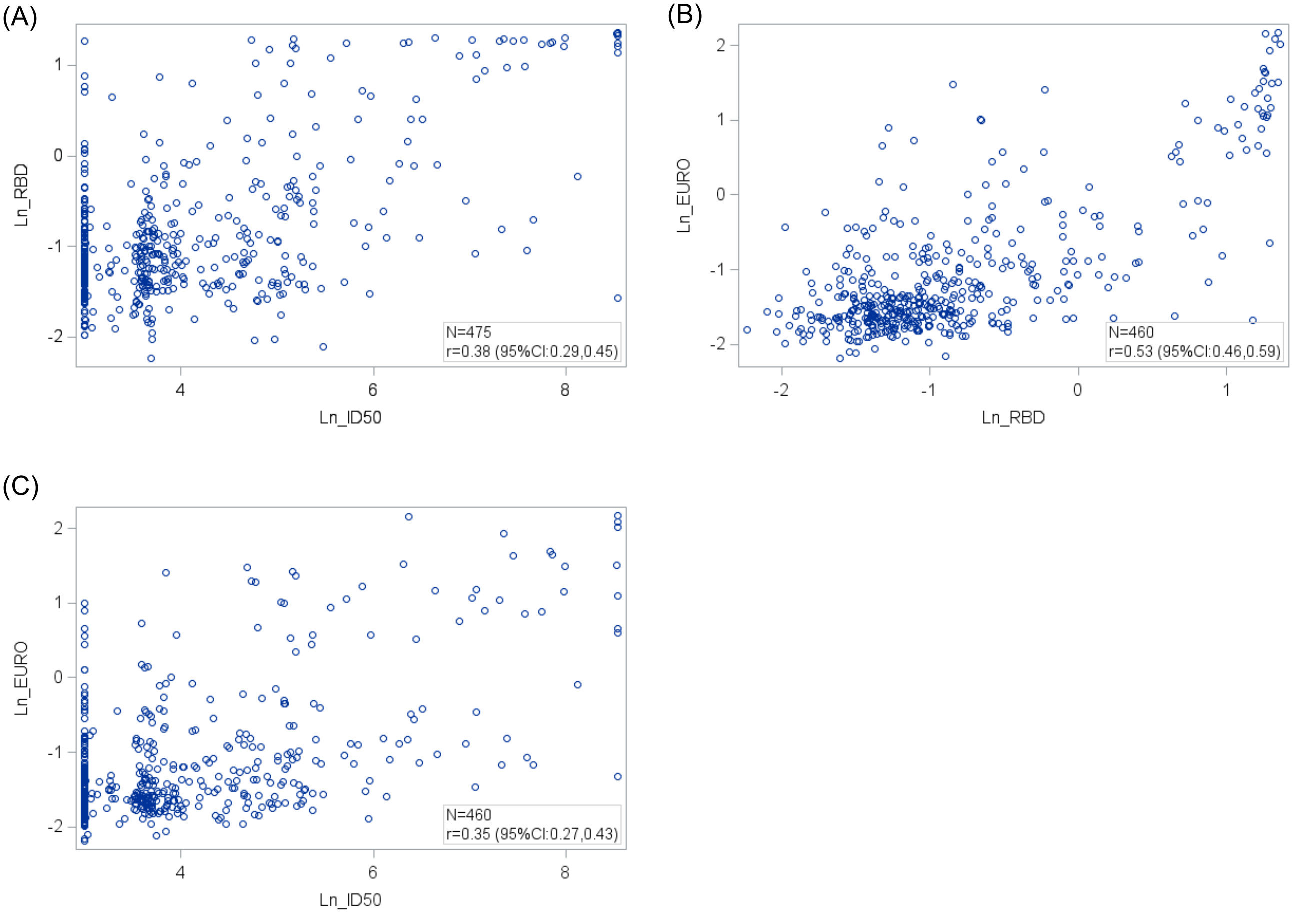

Figure S2. Correlation between RBD binding antibody levels, EUROIMMUN antibody levels, and neutralization titer. RBD binding antibody level and neutralization titer were available on baseline samples from 475 study participants. The EUROIMMUN had baseline samples for 460 participants. Spearman correlations with 95% confidence were calculated using the natural log values of the assays: (A) RBD binding assay and ID50 titer r=0.38 (95% CI: 0.29, 0.45); (B) EUROIMMUN assay and RBD r=0.53 (95% CI: 0.46, 0.59); and (C) EUROIMMUNE assay and ID50 titer r=0.35 (95%CI: 0.27, 0.43).

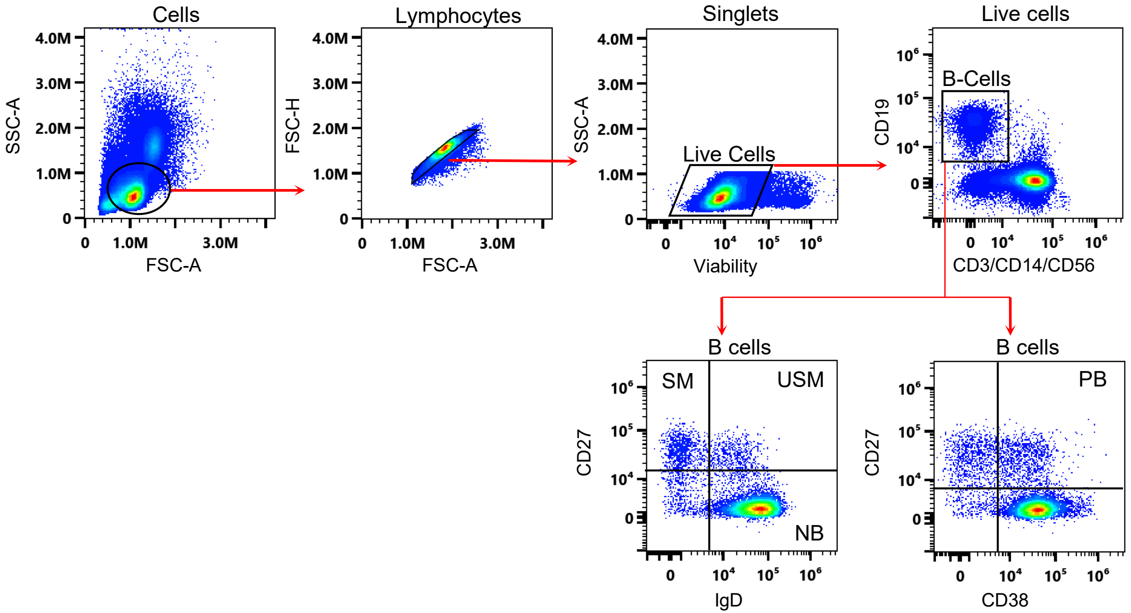

Figure S3. B cell gating strategy.
Representative plots demonstrate gating strategies for B cell subtypes. PB: Plasmablasts, SM: Switched Memory B cells, USM: Unswitched Memory B cells, NB: Naïve B cells.

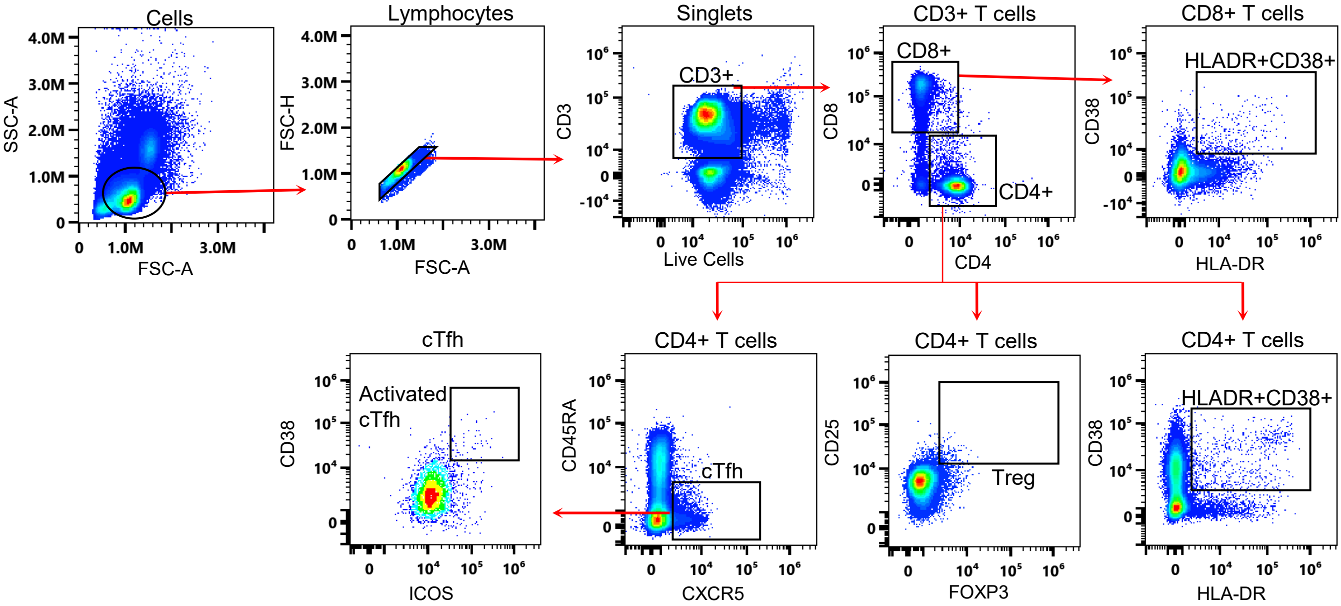

Figure S4. T cell phenotype gating strategy.
Representative plots demonstrate gating strategies for T cell subtypes. Treg: T regulatory cells, cTfh: circulatory T follicular helper cells

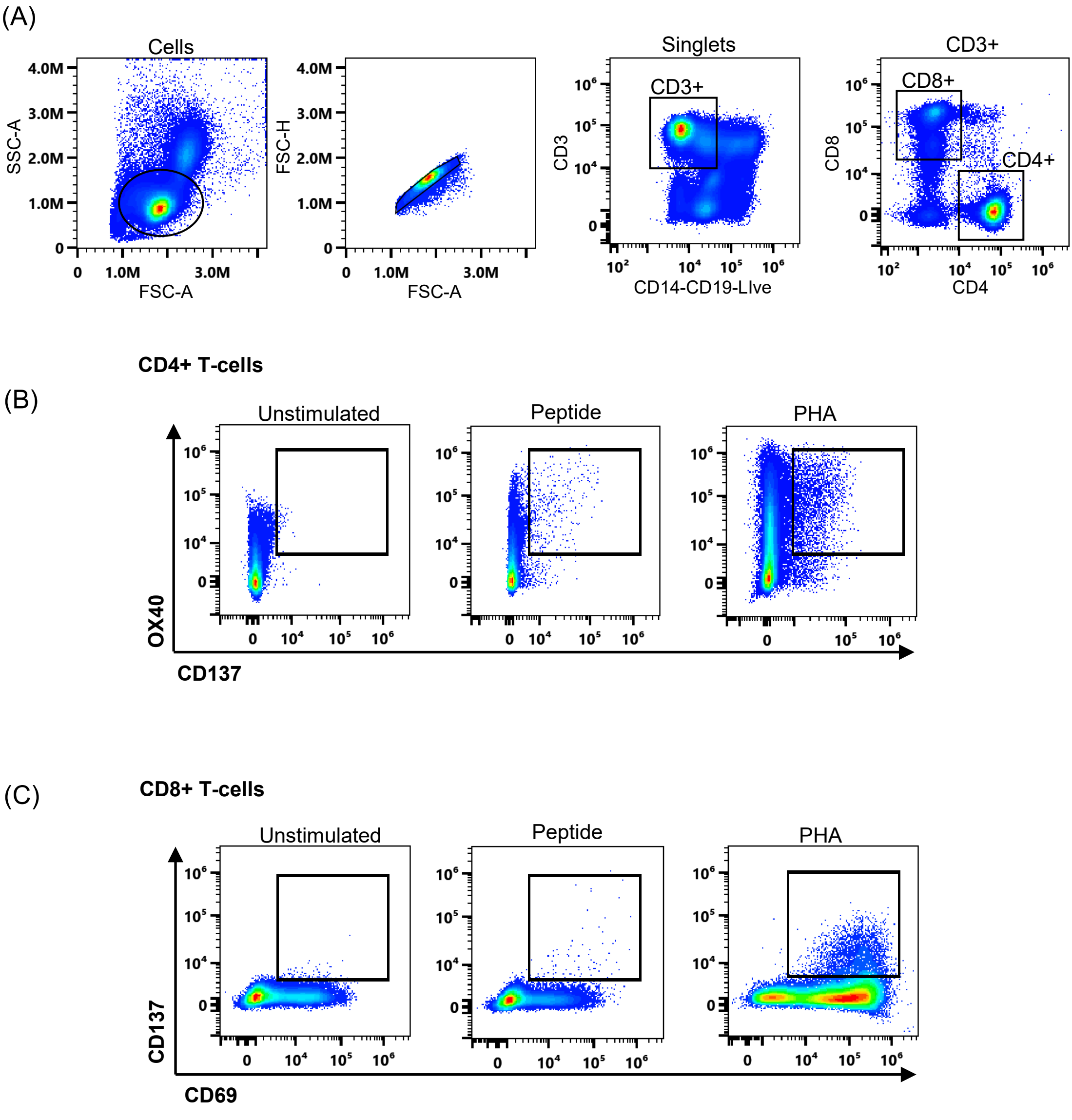

Figure S5. Gating strategy for antigen-specific T cells.
(A) Representative gating strategy for T-cells. (B) Representative plots for unstimulated, SARS-CoV-2 spike peptide- and PHA-stimulated CD4+ T-cells and (C) CD8+ T-cells.

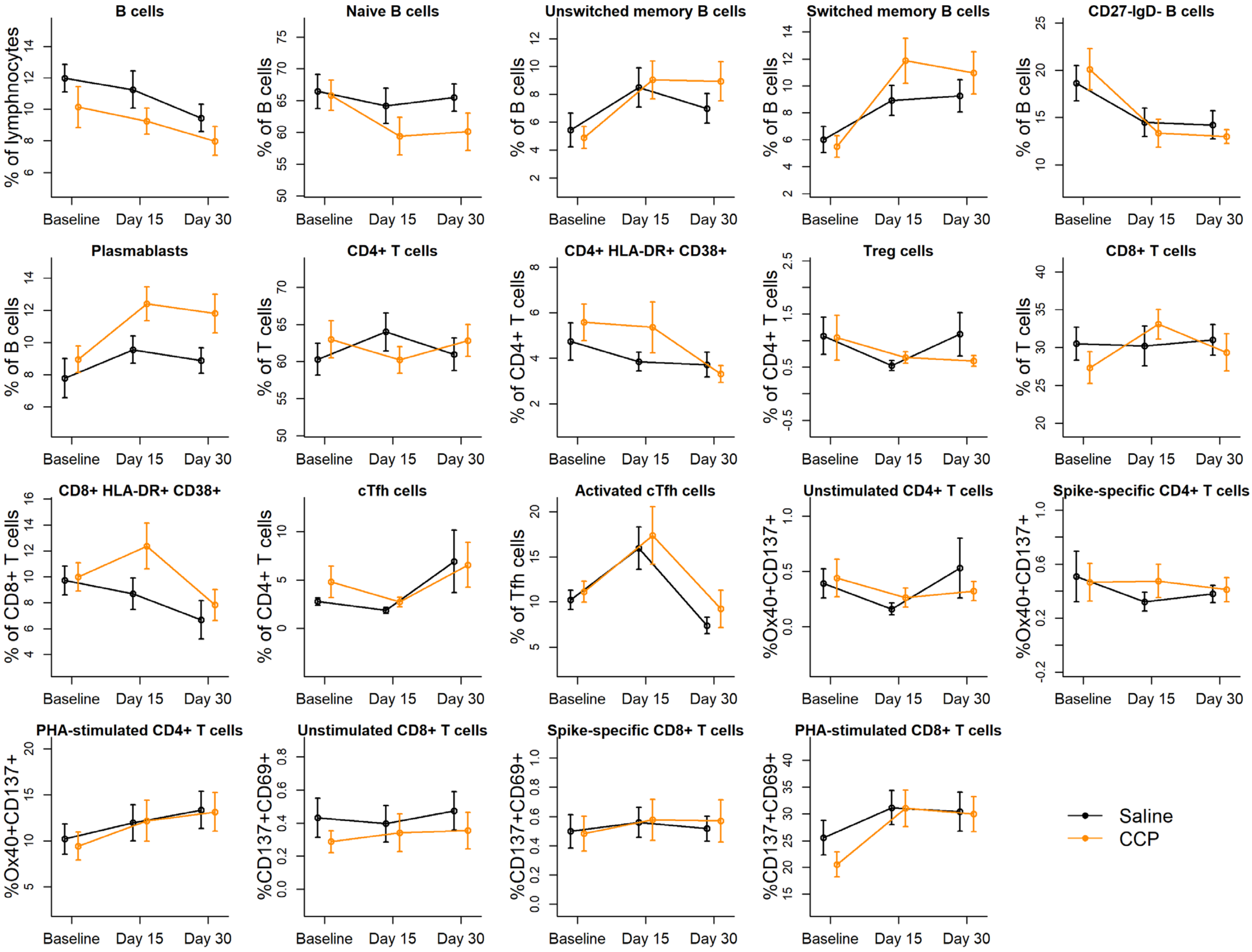

Figure S6. Effect of CCP on cellular immune parameters.
The evolution of each of the cellular phenotypes measured in this study are plotted separated by recipients of saline (black line) or CCP (orange line). Results were analyzed using a linear mixed effects model, and no significant differences in cellular phenotypes were seen between the two groups.
